# Transformer fault diagnosis method based on SMOTE and NGO-GBDT

**DOI:** 10.1038/s41598-024-57509-w

**Published:** 2024-03-26

**Authors:** Li-zhong Wang, Jian-fei Chi, Ye-qiang Ding, Hai-yan Yao, Qiang Guo, Hai-qi Yang

**Affiliations:** 1 State Grid Zhejiang Power Co., Ltd, Hangzhou Linping Power Supply Company, Hangzhou, 311199 China; 2 Hangzhou Electric Power Equipment Manufacturing Co., Ltd, Yuhang Qunli Complete Sets Electricity Manufacturing Branch Electric, Hangzhou, 311000 China; 3 School of Mechanical Engineering, Northeast Electric Power University, Jilin, 132012 China

**Keywords:** Fault diagnosis, Transformers, Oversampling, LightGBM feature selection, GBDT, Northern goshawk optimization algorithm, Electrical and electronic engineering, Power distribution, Power stations

## Abstract

In order to improve the accuracy of transformer fault diagnosis and improve the influence of unbalanced samples on the low accuracy of model identification caused by insufficient model training, this paper proposes a transformer fault diagnosis method based on SMOTE and NGO-GBDT. Firstly, the Synthetic Minority Over-sampling Technique (SMOTE) was used to expand the minority samples. Secondly, the non-coding ratio method was used to construct multi-dimensional feature parameters, and the Light Gradient Boosting Machine (LightGBM) feature optimization strategy was introduced to screen the optimal feature subset. Finally, Northern Goshawk Optimization (NGO) algorithm was used to optimize the parameters of Gradient Boosting Decision Tree (GBDT), and then the transformer fault diagnosis was realized. The results show that the proposed method can reduce the misjudgment of minority samples. Compared with other integrated models, the proposed method has high fault identification accuracy, low misjudgment rate and stable performance.

## Introduction

Power transformers are key equipment in the transmission and transformation system, and their operating status is related to the stability of the power system. When a transformer malfunctions, if accurate diagnosis cannot be made in a timely manner, it will cause significant economic losses. Therefore, how to improve the accuracy of transformer fault diagnosis has always been a hot topic for scholars to study.

As the aging process of transformer insulation progresses, H_2_, CH_4_, C_2_H_6_, C_2_H_4_, C_2_H_2_, CO_2_, and other gases are produced and dissolve into the insulating oil. The present condition of the transformer may be inferred from the concentration and composition of these dissolved gases within the oil^1^. The predominant analytical techniques employed to assess the transformer’s condition encompass the IEC three-ratio method^2^, Rogers’ four-ratio method^3^, Duval Pentagon^4^, Doernberg’s ratio method^5^, among others. In^6^, a fuzzy logic approach was proposed to overcome the shortcomings of traditional IEC methods and enhance the accuracy of model diagnosis. In^7^, based upon the data of dissolved gases within oil, a fuzzy logic-based transformer fault diagnosis model employing the Rogers Four Ratio Method has been developed. The model's implementation has demonstrated its capacity to rectify the deficiencies inherent in conventional fault diagnosis methods, thereby enhancing the accuracy of fault diagnosis. Conversely, this method lacks comprehensive coding and the diagnostic threshold is too rigidly defined, thereby failing to capture the intricate nature of faults within the transformer and compromising the accuracy of fault diagnosis^8^. In^9^, the ratio coding method and raw gas data are used to construct 24-dimensional features, which improves the model's ability to distinguish between different faults and makes it more versatile. Ref.^10^. proposes a PSO-RF diagnostic model that extracts transformer fault characteristic information without using coding ratios, thereby improving the model's fault diagnosis capabilities. However, in existing research, the dimensionality explosion problem is less considered when constructing feature parameters. Because as the sample size increases, the fault diagnosis model becomes better. However, the increase in feature dimension leads to an exponential increase in the amount of calculation and an increase in redundant information. Therefore, it is necessary to remove redundant information to improve model operation efficiency and diagnostic accuracy.

As artificial intelligence technology advances, machine learning applications in transformer fault diagnosis have gained momentum. Support Vector Machine^11–13^, Convolutional Neural Network(CNN)^14,15^, Self-Organizing Mapping Neural Network(SOM)^16^, Gate Recurrent Unit(GRU)^17,18^, Cloud Model(CM)^19^, Adaptive Boosting(AdaBoost)^20^, Gradient Boosting Decision Tree(GBDT)^21^ and other models have demonstrated remarkable success in classification identification. Yet, The fault diagnosis models mentioned above were all constructed based on the assumption of having a relatively large dataset. However, in practical operations, transformers rarely experience failures and the frequencies of different types of faults vary significantly. This makes it difficult to meet the precision requirements using big data samples. Therefore, when addressing the practical challenges of transformer fault diagnosis, the issue of sample imbalance needs to be given immediate attention in order to achieve precision.

The formulation of transformer fault diagnosis models hinges upon an abundance of data sets. In practical operations, the likelihood of transformer malfunction is slim; the variance of diverse fault types is vast, thereby making it challenging to attain the requisite standards for extensive datasets.

Research on imbalanced datasets mainly focuses on developing classifiers and data preprocessing techniques. Data-level processing involves reconstructing the dataset to better align with its inherent characteristics, thereby addressing issues arising from an imbalance in sampling frequency. undersampling^22^ involves selecting a subset of the most representative samples from the majority classes to mitigate the issue of class imbalance. However, this approach may result in the loss of crucial information regarding the bulk of sample classes, ultimately impairing the performance of classifiers. Oversampling involves artificially increasing a limited sample size to achieve data balance. This can be done through techniques such as Synthetic Minority Oversampling Technique(SMOTE)^23,24^, SVM SMOTE^25^, Borderline-SMOTE^26^, Adaptive Synthetic Sampling(ADASYN)^27^, Generative Adversarial Network(GAN)^28^, and others. Common approaches at the classification algorithm level include CostSensitive^29^ and Ensemble Learning^30^. In^31^, cost-sensitive classifiers are used to address class disparities and improve fault categorization accuracy. The Auxiliary Generation Mutual Countermeasure Network (AGMAN) was proposed in Ref.^32^. to enhance the accuracy of small sample class imbalance fault diagnosis. In^33^, MeanRadius-SMOTE is proposed based on the traditional SMOTE oversampling algorithm, which effectively avoids the generation of useless samples and noisy samples, and the generalization of this algorithm is verified.

The main contributions of this work are as follows: (1) Improved classification performance on imbalanced and small sample data using oversampling methods, avoiding classifiers focusing too much on majority samples and causing the classifier's hyperplane to shift towards minority class samples. (2) Established a deep relationship between dissolved gases in oil and fault types, reduced redundancy between features by using LightGBM feature selection, and improved the computational efficiency of the diagnostic model. (3) Optimized algorithm for parameter optimization of the diagnostic model to establish the optimal diagnostic model. Finally, the effectiveness of the proposed methods in this paper was verified through different sampling methods, different feature selection methods, and different diagnostic models.

## NGO-GBDT transformer fault diagnosis method based on balanced data set

### Composite minority oversampling technique

The main idea of the SMOTE is to randomly select a majority class sample, then find the *k* nearest neighbors, and select a sample from the *k* nearest neighbors according to the sampling probability to generate a new sample based on formula (1), repeatedly balancing the dataset.

1 $$ Y = Z_{i} + rand \times \left( {Z_{1} - Z_{2} } \right) $$

Among them, *Z*_*1*_ is the majority class sample; *Z*_*2*_ is one of the *k* samples closest to *Z*_*i*_; rand belongs to a random number of [0,1]; *Y* represents the newly generated minority class sample.

### Northern goshawk optimization algorithm

The northern goshawk optimization algorithm^34^ is a new meta heuristic algorithm proposed in 2022, which simulates the behavior of northern goshawks during hunting. The hunting strategy is mainly divided into two stages: prey identification stage and chase and escape behavior stage.

1. Initialization phase
  Population initialization, as shown in formula (2):
  2 $$ X = \left( \begin{gathered} X_{1} \\ \vdots \\ X_{i} \\ \vdots \\ X_{N} \\ \end{gathered} \right)_{N \times m} = \left( {\begin{array}{*{20}c} {x_{1,1} } & \cdots & {x_{1,j} } & \cdots & {x_{1,m} } \\ \vdots & \ddots & \vdots & {\mathinner{\mkern2mu\raise1pt\hbox{.}\mkern2mu \raise4pt\hbox{.}\mkern2mu\raise7pt\hbox{.}\mkern1mu}} & \vdots \\ {x_{i,1} } & \cdots & {x_{i,j} } & \cdots & {x_{i,m} } \\ \vdots & \ddots & \vdots & \ddots & \vdots \\ {x_{N,1} } & \cdots & {x_{N,j} } & \cdots & {x_{N,m} } \\ \end{array} } \right) $$
  Among them, *X* represents the matrix of the population, *X*_*i*_ is the initial value of the *i*th individual, *x*_*i,j*_ are the values of the jth dimension of the *i*th individual, *N* is the number of populations, and *m* is the dimension of the search space.
  The objective function of the population is shown in formula (3):
  3 $$ F(X) = \left( \begin{gathered} F_{1} = F(X_{1} ) \\ \vdots \\ F_{i} = F(X_{i} ) \\ \vdots \\ F_{N} = F(X_{N} ) \\ \end{gathered} \right)_{N \times 1} $$
  Among them, *F* is the vector of the obtained objective function value, and *F*_*i*_ is the objective function value corresponding to the *i*th solution.
2. Prey identification stage
  In the first stage of hunting, the goshawk randomly selects its prey and quickly attacks it. The mathematical expressions of the northern goshawk at this stage are shown in formulas (4) to (6):
  4 $$ P_{i} = X_{k} ,k = 1,2, \cdots ,k - 1, \cdots ,N $$
  5 $$ x_{x,j}^{new,p1} = \left\{ \begin{gathered} x_{i,j} + r\left( {p_{i,j} - Ix_{i,j} } \right),F_{p,i} < F_{i} \hfill \\ x_{i,j} + r\left( {x_{i,j} - p_{i,j} } \right),F_{p,i} \ge F \hfill \\ \end{gathered} \right. $$
  6 $$ X_{i} = \left\{ \begin{gathered} X_{i}^{new,p1} ,{\text{F}}_{i}^{new,p1} < F_{i} \hfill \\ {\text{X}}_{{\text{i}}} ,{\text{F}}_{i}^{new,p1} \ge F \hfill \\ \end{gathered} \right. $$
  Among them, *P*_*i*_ is the prey position corresponding to the *i*th goshawk; *F*_*p,i*_ is the corresponding objective function value; A random integer* k* $\in$[1, N] and not equal to i; *xnew,p1 x,j* are the new positions of the *i*th solution, and *Fnew,p1 i* are the corresponding objective function values for the prey recognition stage; random numbers with *k* $\in$[0,1]; *I* = 1 or 2; *r* and *I* are random numbers used to generate random NGO behavior in search and update.
3. Chasing and escaping behavior stage
  After attacking its prey, the eagle instinctively attempts to escape. Due to the rapid and agile movements of the goshawk, it pursues its prey in any situation and ultimately hunts. The mathematical expressions at this stage are as follows:
  7 $$ x_{x,j}^{new,p2} = x_{i,j} + R\left( {2r - 1} \right)x_{i,j} $$
  8 $$ R = 0.02\left( {1 - \frac{t}{T}} \right) $$
  9 $$ X_{i} = \left\{ \begin{gathered} X_{i}^{new,p1} ,{\text{F}}_{i}^{new,p2} < F_{i} \hfill \\ {\text{X}}_{{\text{i}}} ,{\text{F}}_{i}^{new,p2} \ge F \hfill \\ \end{gathered} \right. $$
  where: *t* and *T* represent the current and maximum number of iterations, respectively; *R* is the attack radius, which decreases as the number of iterations increases; *Xnew, p2 x, j* are the new positions of the *i*th solution; *Fnew,p2 i*is the objective function value for this stage.

### Based on LightGBM feature selection

LightGBM^35^ is an efficient framework of gradient enhancement decision tree algorithm, which can evaluate the importance of features, and speed up the training of models by eliminating features of low importance to avoid dimensional disasters. The steps for calculating the feature importance are as follows:

For the training set *W* = {w_1_,w_2_,…,w_n_} corresponding to{g_1_,g_2_,…,g_n_}, the sampling rate of the sample is *a*, and the sampling rate of the small gradient sample is b, then the steps for calculating feature importance are as follows:

Step 1: sorting the fault samples in descending order according to the absolute value of the gradient;

Step 2: selecting the initial *a* × *N* samples to form a large gradient sample subset *C*_*1*_;

Step 3: a random selection of *b* × *N* samples is drawn from the remaining faulty samples to form a smaller gradient sample subset *D*_*1*_;

Step 4: adopting *C*_*1*_ × *D*_*1*_ to learn new decision trees, assign weights (1-a)/b to small gradient faulty samples when calculating information gain at computing nodes;

Step 5: reiterate Steps 1–4 until reaching a predetermined iteration count or convergence threshold. Throughout the model, the sum of the information gain of each feature across all nodes of a split feature represents the significance of that feature.

### Transformer fault diagnosis process

**Figure 1:**
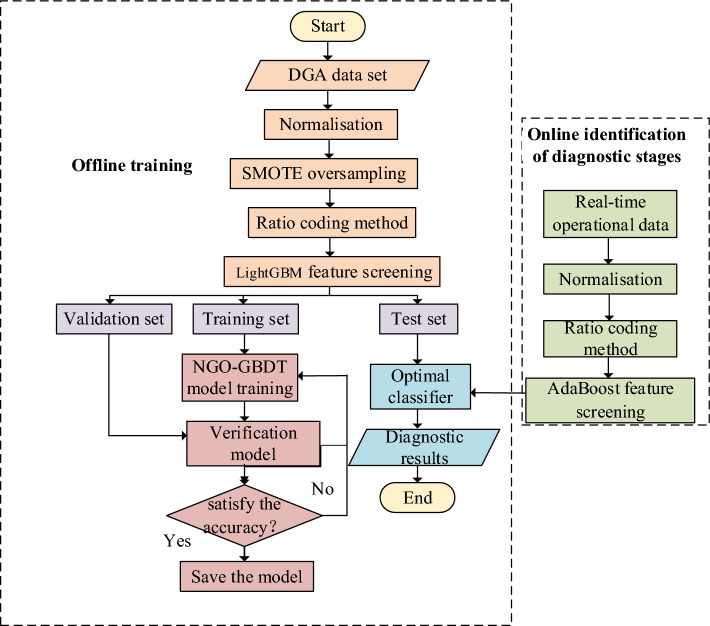
Flow chart of transformer fault diagnosis.

The methodology for transformer fault diagnosis through SMOTE and NGO-GBDT entails two stages: offline model training and online identification and diagnosis. The specific workflow is depicted in Fig. 1. During the offline training stage for transformer faults, a single session suffices; upon securing the optimal diagnostic model, the deployment is conducted, paving the way for online identification and diagnosis.

The specific steps in the offline training phase are as follows:

Step 1: Sample data preprocessing. The collected DGA samples are normalized, and the data set is balanced through the application of the SMOTE.

Step 2: Feature selection. The candidate feature set is established through the code-free ratio method, while the optimal input feature is determined via the LightGBM.

Step 3: Model training and validation. The training set, validation set, and test set are separated; the parameters of the GBDT model, including those of max_ depth, n_ estimators, and learning_rate, are optimized using the NGO algorithm. Therefore, utilizing the verification set to assess the diagnostic efficacy of each iterationary model, if the disparity in accuracy between successive training sessions does not exceed five percent, save the model parameters upon conclusion of the training; In the event that such conditions are not met, one must retrain the model until they are fulfilled.

Step 4: Model validation. The test dataset is fed into the optimal model; the diagnostic accuracy of the NGO-GBDT model is validated.

The specific steps in the online identification and diagnosis stage are as follows:

Step 1: Sample data preprocessing. Normalizing the DGA samples collected in real-time.

Step 2: The candidate feature set is established through the code-free ratio method, while the optimal input feature is determined via the LightGBM.

Step 3: The subset of optimal characteristics is directly inputted into the optimal model, thereby obtaining the results of online diagnosis of transformers.

### Transformer fault diagnosis process

In the context of imbalanced data classification, an overabundance of samples from dominant classes can result in the model’s tendency to excessively focus on the majority categories, thereby neglecting a select few. This can lead to the plane of the classifier shifting towards a subset of samples within these categories. To effectively assess the efficacy of the transformed transformer fault diagnosis model, this paper selects a multi classification evaluation index system based on confusion matrix, with accuracy, recall, F1 value, G-mean, and Kappa coefficient as the model evaluation indicators.

1. Precision and recall
  The accuracy is the proportion of predicted positive samples to actual positive samples. The recall rate represents the proportion of predicted positive samples in the actual positive sample results.
  10 $$ P = \frac{TP}{{TP + FP}} $$
  11 $$ R = \frac{TP}{{TP + FN}} $$
  Among them, represents the accuracy; *R* represents the recall rate; *TP* is the case when the classification of the positive sample is correct; *FP* is the case where the counter example sample is misclassified; *F*_*N*_ is the case where the positive sample is misclassified.
2. F1 value (F1 score)
  The F1 value represents the harmonic average of accuracy and recall.
  12 $$ F1 = \frac{2PR}{{P + R}} $$
3. Kappa coefficient
  The kappa coefficient reflects the consistency between real classification and predicted classification, and is one of the commonly used indicators to evaluate the accuracy of fault diagnosis.
  13 $$ Kappa = \frac{{p_{0} - p_{e} }}{{1 - p_{e} }} $$
  Among them, *P*_0_ is the number of correctly predicted samples divided by the total number of samples.
  Assuming that the true samples for each class are *a*_1_,* a*_2_, …, *a*_*e*_, and the predicted unclassified samples are *b*_1_,* b*_2_, …, *b*_*e*_, respectively
  14 $$ p_{e} = \frac{{a_{1} \times b_{1} + a_{2} \times b_{2} + \cdots + a_{n} \times b_{n} }}{n \times n} $$
  The range of Kappa coefficient values is [0,1], which is generally divided into five groups to represent different levels of consistency: 0–0.20 (extremely low consistency), 0.21–0.40 (general consistency), 0.41–0.60 (medium consistency), 0.61–0.80 (high consistency), and 0.81–10 (almost identical). That is, the closer the kappa coefficient is to 1, the better the diagnostic effect.

## Example analysis

According to DL/T722-2014 Analysis and Judgment Criteria for Dissolved Gases in Transformer Oil^27^, transformers are classified into six types based on whether or not the transformer has malfunctioned and the type of fault. They are represented by labels 1–6, including low energy discharge (D1), high energy discharge (D2), medium low temperature heat release (T1&T2), high temperature heat release (T2), partial discharge (PD), and normal (N) This article selects 480 sets of monitoring data provided by a power supply company in Yuhang City, Zhejiang Province as the fault sample set. Each operating state in the sample set includes 5 characteristic gases, including H_2_, CH_4_, C_2_H_6_, C_2_H_4_, and C_2_H_2_. The distribution of the dataset and sample labels are shown in Table 1.

**Table 1:** Dataset distribution and sample labels.

Status type	Quantity	Proportion (%)	Label
N	78	16.3	1
D2	105	21.9	2
T1&T2	147	30.6	3
T2	76	15.8	4
PD	45	9.4	5
D1	29	6.0	6

### Data preprocessing

When a transformer malfunctions, the composition and content of dissolved gases in the insulation oil will change. This article selects H_2_, CH_4_, C_2_H_6_, C_2_H_4_, and C_2_H_2_ dissolved in the oil as sample inputs. Normalize the characteristic gas, as shown in formula (15):

15 $$ x^{\prime} = \frac{{x_{i} - x_{i\min } }}{{x_{i\max } - x_{i\min } }} $$

Among them, *x*_*i*_ and *x*′ are features before and after normalization; *Ximin* and *Ximax* is the minimum and maximum values of each column feature in the raw data before normalization.

### Data balancing processing

In accordance with Table 1, it becomes apparent that mid-to-low temperature overheat failures constitute 30.6% of all instances, whereas partial discharge failures comprise merely 6.0%. Should the data sets employed for model formulation be imbalanced, the model may not acquire sufficient proficiency in certain sample types, leading to an increased likelihood of misclassifying these sample types during the identification stage, thereby compromising the accuracy of the model's classification. In this study, we employ SMOTE to balance the dataset. The sample distribution subsequent to SMOTE oversampling is depicted in Table 2. In preparation for subsequent feature optimization, model training, and diagnostic purposes, the sample count for each category in Table 2 has been harmonized.

**Table 2:** Data distribution before and after SMOTE balancing.

Data type	Sample quantity					
	N	D2	T1&T2	T2	PD	D1
Raw data	78	105	147	76	45	29
After SMOT balancing treatment	147	147	147	147	147	147

### Optimization of transformer fault characteristics

In the field of DGA fault diagnosis, the IEC three-ratio method, Rogers four-ratio method, and uncoded ratio method are generally used as references. However, the above methods have incomplete feature selection and insufficient data utilization, and cannot fully reflect the relationship between faults and features. Therefore, this paper uses 5 characteristic gases as the basis to construct 19-dimensional ratio characteristics, as shown in Table 3.

**Table 3:** Characteristic code and characteristic quantity of dissolved gas in oil.

Feature encoding	Characteristic quantity	Feature encoding	Characteristic quantity
S1	H_2_	S11	CH_4_/H_2_
S2	CH_4_	S12	C_2_H_2_/H_2_
S3	C_2_H_6_	S13	C_2_H_4_/THC
S4	C_2_H_4_	S14	C_2_H_6_/THC
S5	C_2_H_2_	S15	CH_4_/THC
S6	CH_4_/ C_2_H_6_	S16	C_2_H_2_/THC
S7	CH_4_/ C_2_H_4_	S17	H_2_/THC
S8	C_2_H_6_/ C_2_H_4_	S18	(CH_4_+ C_2_H_4_)/THC
S9	C_2_H_2_/ C_2_H_4_	S19	H_2_/ALL
S10	C_2_H_4_/ C_2_H_6_		

THC = CH_4_+ C_2_H_6_+ C_2_H_4_+ C_2_H_2_.

ALL = H_2_+ CH_4_+ C_2_H_6_+ C_2_H_4_+ C_2_H_2_.

**Figure 2:**
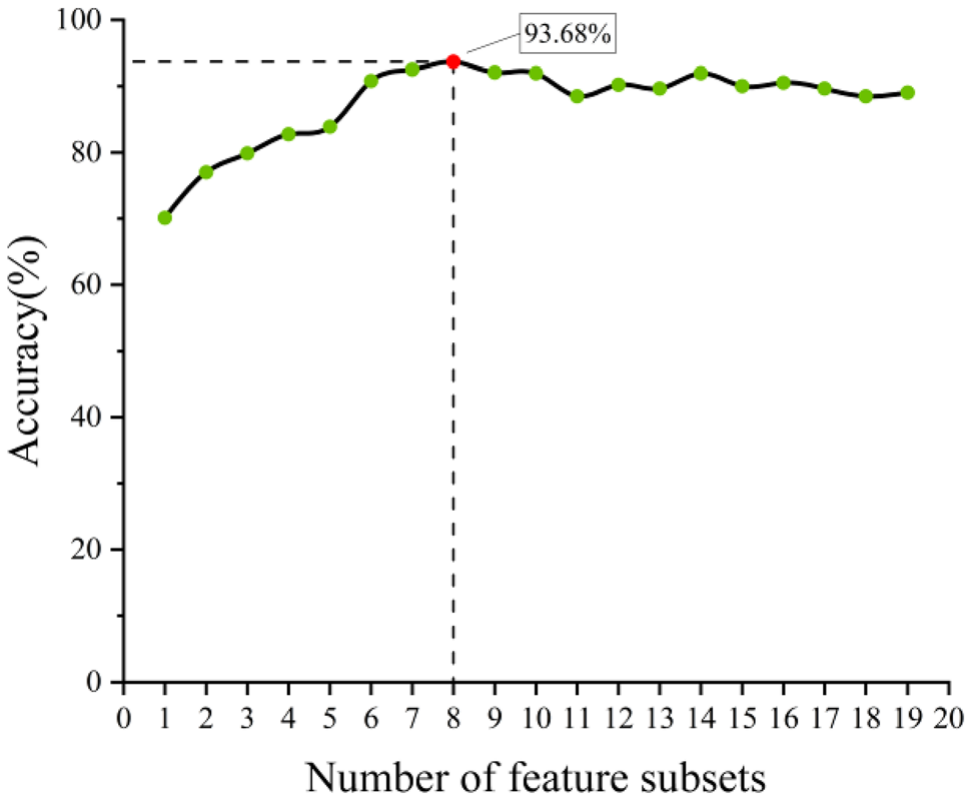
The number of feature subsets corresponds to the average diagnostic accuracy of the model.

The 19-dimensional features constructed in this article expand the feature space and make full use of data information. However, there will be information redundancy. These redundant features will increase the computational burden of the model. It is necessary to reduce the data dimensions and reduce the complexity of the model. Therefore, the LightGBM feature importance evaluation method is introduced to optimize the 19-dimensional features. The feature importance ranking results are shown in Table 4. Features sorted according to the importance of LightGBM features are sequentially and incrementally input into the GBDT model for diagnosis and identification. In order to avoid contingency, ten-fold cross-validation is performed on the input data sampling, and the average accuracy is taken as the final result, as shown in Fig. 2. As the number of features increases from 1 to 8 in Fig. 2, the diagnostic accuracy of the GBDT model gradually increases. When the number of features is 8, the average diagnostic accuracy reaches a maximum of 93.68%. When the fault diagnosis accuracy reaches a high point, as the number of features continues to increase, its accuracy remains unchanged or decreases. The reason is that too many features lead to an increase in the complexity of the model. Based on this, the first 8-dimensional features sorted by LightGBM are selected for model training and diagnosis.

**Table 4:** Feature importance ranking results.

Feature ranking	Feature encoding value (%)	Feature encoding	Feature ranking	Feature encoding value (%)	Feature encoding
1	18.7	S11	11	3.0	S19
2	15.0	S3	12	2.7	S17
3	14.2	S7	13	2.1	S18
4	8.7	S9	14	1.8	S8
5	5.5	S1	15	1.7	S10
6	5.0	S4	16	1.4	S14
7	4.4	S5	17	0.4	S5
8	4.0	S12	18	0.9	S15
9	4.0	S2	19	0.8	S6
10	3.5	S16

### Analysis of fault diagnosis results

The selected optimal feature subset is divided into training set, test set and verification set according to the ratio of 6:2:2. The specific distribution is shown in Table 5.

**Table 5:** Distribution of the sample data.

Status type	Training set	Test set	Verification set
N	89	29	29
D2	89	29	29
T1&T2	89	29	29
T2	89	29	29
PD	89	29	29
D1	89	29	29

**Figure 3:**
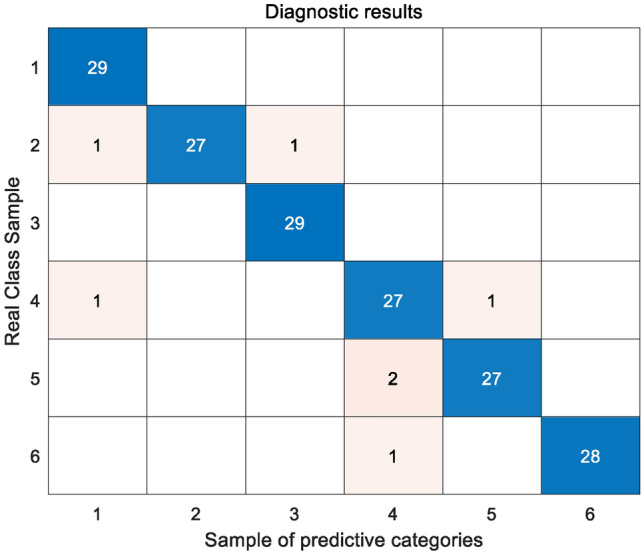
Test set sample confusion matrix.

In order to ensure the accuracy and effectiveness of the model, NGO is used to optimize max_depth, learning rate, learning_rate and n_estimators. The GBDT hyperparameter optimization range is set as shown in Table 6. Figure 3 shows the confusion matrix of the transformer fault diagnosis results based on SMOTE and NGO-GBDT. The blue diagonal line in the figure represents the number of correct predictions in the real samples, and the sum of each row of data is expressed as the total number of samples. Among the 174 test samples in Fig. 3, a total of seven fault samples were misjudged. The total accuracy of transformer fault diagnosis was 95.98%. Among them, normal and medium–low temperature exothermic samples were correctly identified. Among them, the misjudgment rates for high-temperature exothermic, low-energy discharge and partial discharge samples are only 7.40%, 7.40% and 3.45%, indicating that the model proposed in the article has good stability. Based on the information in the confusion matrix, the precision P, recall R and F1 values of the diagnostic model are 0.9598, 0.9601 and 0.9599 respectively. The Kappa coefficient of the model is 0.9521, that is, the consistency between the model's true classification and the predicted classification is almost completely consistent, indicating that The model proposed in this article has strong fault identification and classification capabilities.

**Table 6:** Hyperparameter optimization range setting.

Hyperparameter name	Optimization scope
Max_depth	(2,10)
Learnning_rate	(0.1,0.5)
n_estimators	(0,50)

## Results and discussion

### Comparative analysis of different feature selection methods

**Figure 4:**
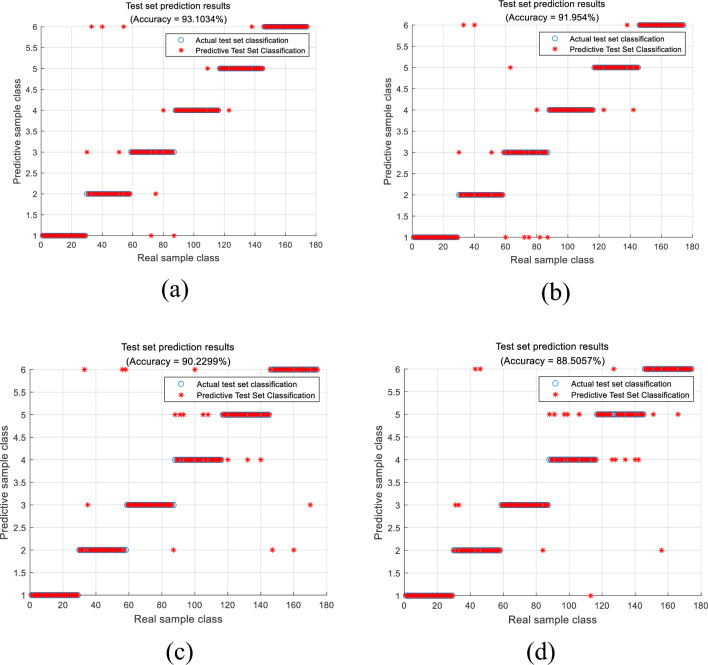
Comparison of diagnostic results of different feature optimization methods. (**a**) Recursive feature elimination, (**b**) RF feature selection, (**c**) XGBoost feature selection, (**d**) 19 dimensional joint features.

To validate the efficacy of the proposed feature selection strategy, this paper employs four distinct approaches: Recursive Feature Elimination (RFE), XGBoost Feature Selection, RF Feature Optimization, and 19-dimensional feature extraction as inputs for the NGO–GBDT model. The classification results are delineated in Fig. 4 and Table 7. It is apparent from Table 7 that, following the rigorous selection of features, the diagnostic precision and Kappa coefficient undergo significant enhancement across various degrees, while the duration of operation diminishes. Among these methods, the LightGBM feature selection approach exhibits the most favorable diagnostic performance compared to the others, thereby affirming the superiority of the LightGBM feature selection method.

**Table 7:** The Kappa coefficient of different features was selected for comparison.

Feature subset type	Kappa coefficient	Calculation time (s)
LightGBM feature selection	0.9521	0.257
RFE	0.9183	0.486
RF feature selection	0.9000	0.512
XGBoost feature selection	0.9850	0.663
19 dimensional features	0.8652	1.075

### Comparative analysis of sample equalization effects

**Figure 5:**
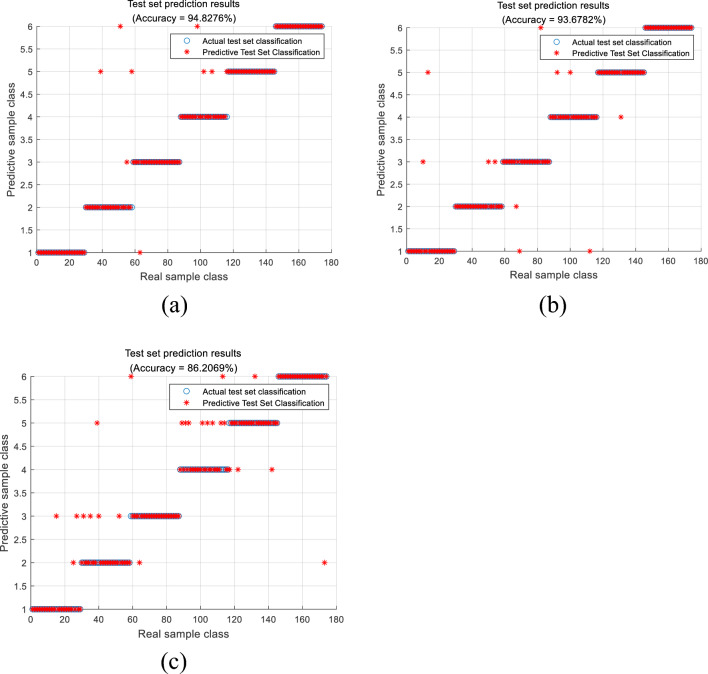
Comparison of sample equalized diagnosis (**a**) ADASYN oversampling, (**b**) random oversampling, (**c**) imbalanced dataset.

In order to verify the effectiveness of the diagnostic model in processing unbalanced data, random oversampling and ADASYN oversampling methods were used from the data processing level to compare the diagnostic results with the original data set. The confusion matrix is shown in Fig. 5, and the model evaluation indicators are shown in Table 8 shown. According to the diagnosis results, it can be seen that the original data set without balance processing has insufficient training for minority class samples, resulting in high misjudgment rates for the three types of minority class samples: high temperature overheating, partial discharge, and low energy discharge during identification and diagnosis. After oversampling, the model diagnosis accuracy and Kappa coefficient have been improved to varying degrees. After using SMOTE for data enhancement in this article, the diagnostic accuracy and comprehensive indicators of each type are better than other sampling methods, further validating this article. The superiority of the proposed method in handling imbalanced data.

**Table 8:** Comparison of diagnostic results under different sampling methods.

Data sampling method	Accuracy	Kappa coefficient
ADASYN oversampling	94.83%	0.9386
Random oversampling	93.68%	0.9251
Imbalanced dataset	86.21%	0.8360

### Comparative analysis of diagnostic effects of multiple models

In order to verify that the integrated learning method proposed in this article can effectively improve the accuracy of transformer fault identification, NGO-GBDT was compared with WOA-GBDT, GBDT, RF and DT, and the model classification effect was evaluated through multiple indicators. In order to make the model more convincing, the GA-XGBoost diagnostic model proposed in Ref.^36^. and The PSO-BiLSTM diagnostic model proposed in Ref.^37^. The WOA-SVM diagnostic model proposed in^38^ ensures that the input features are consistent. Table 9 shows the diagnostic results of different models.

**Table 9:** Model comparison analysis results.

Model name	Precision	Recall	F1 value	Kappa coefficient	Accuracy (%)
NGO-GBDT	0.9598	0.9601	0.9599	0.9521	95.98
GA-XGBoost	0.9427	0.9440	0.9433	0.9417	94.30
PSO-BiLSTM	0.9372	0.9352	0.9347	0.9352	93.66
WOA-SVM	0.9298	0.9308	0.9304	0.9288	92.32
WOA-GBDT	0.9540	0.9573	0.9556	0.9453	94.53
GBDT	0.9425	0.9425	0.9425	0.9318	93.18
RF	0.9253	0.9269	0.9261	0.9117	91.17
DT	0.9138	0.9193	0.9165	0.8983	89.83

From the perspective of a single diagnostic model, GBDT has a better classification effect than RF and DT. After optimizing the hyperparameters of the GBDT model through the optimization algorithm, the model diagnosis accuracy has been improved, indicating that the NGO optimization algorithm has strong optimization capabilities. It can effectively improve model diagnosis performance. At the same time, comparing GA-XGBoost, PSO-BiLSTM and WOA-SVM with NGO-GBDT, the diagnostic accuracy increased by 1.68%, 2.32% and 3.66% respectively. Based on the parameter analysis of recall rate, precision rate, F1 value and Kappa coefficient, it is shown that the model proposed in this article has better diagnostic effect than other models, verifying the superiority of the NGO-GBDT model.

## Conclusion

In response to the issue of misjudgment of minority samples caused by imbalanced transformer fault samples, this paper proposes a transformer fault diagnosis method based on SMOTE and NGO-GBDT based on data oversampling and ensemble learning algorithm models. The following conclusions are drawn from actual data:

1. By using the LightGBM feature selection method to select the optimal feature subset, redundant information can be avoided and the accuracy of transformer fault identification can be effectively improved.
2. This article deals with imbalanced fault samples from the data processing level, and solves the problem of low diagnostic accuracy caused by insufficient and imbalanced sample data through the SMOTE oversampling method, reducing the misdiagnosis rate of the diagnostic model.
3. Compared with other ensemble learning models, this article constructs an NGO–GBDT transformer fault diagnosis model with high diagnostic accuracy, and further verifies the superiority of the proposed method through evaluation indicators such as accuracy, recall, F1 value, etc.

In summary, the strategy proposed in this paper enables the online diagnosis of electrical transformers, augmenting the operational efficiency of transformer management; to some extent, addressing the scarcity and imbalance of fault sample occurrence during actual operation. Yet, in the selection of K near-neighbors for the synthesis of new samples, this approach possesses a certain degree of blindness, subject to interference from noisy samples, and lacks clarity regarding the boundary between samples, hindering the model's diagnostic capabilities. The text insufficiently delves into the study of dissolved gases in oil, neglecting the impact of two distinctive gases—CO and CO_2_—on transformer faults. Further research is imperative to thoroughly analyze and enhance these issues.

## Data Availability

The datasets generated and/or analysed during the current study are not publicly available due [The data set is a company secret] but are available from the corresponding author on reasonable request. E-mail:xhaiqi0526@163.com.
